# Persistence of Virus-Reactive Serum Immunoglobulin M Antibody in Confirmed West Nile Virus Encephalitis Cases

**DOI:** 10.3201/eid0903.020531

**Published:** 2003-03

**Authors:** John T. Roehrig, Denis Nash, Beth Maldin, Anne Labowitz, Denise A. Martin, Robert S. Lanciotti, Grant L. Campbell

**Affiliations:** * Centers for Disease Control and Prevention, Fort Collins, Colorado, USA; † New York City Department of Health, New York, New York, USA

**Keywords:** IgM, West Nile virus, serology, MAC-ELISA, dispatch

## Abstract

Twenty-nine laboratory-confirmed West Nile virus (WNV) encephalitis patients were bled serially so that WNV-reactive immunoglobulin (Ig) M activity could be determined. Of those patients bled, 7 (60%) of 12 had anti-WNV IgM at approximately 500 days after onset. Clinicians should be cautious when interpreting serologic results from early season WNV IgM-positive patients.

In late summer and early fall of 1999, human West Nile virus (WNV) infections were recognized for the first time in the Western Hemisphere (*1*–*6*). Since its original introduction into the New York City (NYC) area, WNV has caused disease in humans, horses, and a wide variety of birds and other vertebrates, spreading into the eastern two thirds of the United States and also into Canada and the Caribbean Basin (*7*,*8*). The apparent ability of WNV to be disseminated by infected birds and to persist from year to year indicates that it will continue to be a public health problem for the foreseeable future.

The detection of human cases of WNV encephalitis early in the transmission season is a valuable tool to identify human risk and seasonal virus activity. Diagnosis of WNV encephalitis is made by using an immunoglobulin (Ig) M antibody capture enzyme-linked immunosorbent assay (MAC-ELISA), which demonstrates virus-reactive IgM in serum or cerebrospinal fluid (CSF) from a person with a clinically compatible illness (*6*,*9*–*16*). However, if WNV-reactive IgM is long lasting (e.g., from one transmission season to the next), an early season positive IgM test could result from either a recent or past infection.

At least four previous studies, three with Japanese encephalitis virus (JEV) (*13*,*17*,*18*) and one with WNV (*19*), included evaluations of flavivirus-reactive IgM persistence. Only one of the JEV studies evaluated the IgM activity in case-patients >6 months after illness (*17*). Three of 41 JEV case-patients demonstrated JEV-reactive IgM in serum >10 months (300, 330, and 350 days) after onset. In the latter study of human WNV infections, 50% of patients were still IgM positive after 2 months (*19*). We report the results of a longitudinal study that followed laboratory-confirmed human WNV encephalitis case-patients for up to 18 months to determine the longevity of their serum WNV-reactive IgM.

## The Study

Of the 55 surviving laboratory-confirmed case-patients diagnosed with WNV encephalitis in the United States in 1999 (*6*), 29 agreed to participate in follow-up studies to assess their recovery from disease and their WNV-reactive antibody levels. Serum specimens were obtained and analyzed for the presence of anti-WNV IgM and IgG antibodies by using MAC-ELISA and indirect IgG ELISA as described (*16*,*20*). All cases were originally laboratory confirmed by presence of WNV-reactive IgM in acute cerebral spinal fluid specimens, identification of WNV-reactive IgM in serum samples in the presence of WNV-specific neutralizing antibodies, a >4-fold increase in WNV-reactive neutralizing antibodies in serial serum specimens, or a combination of these antibody activities.

For this analysis, all serum specimens from these case-patients were considered independent samples, and multiple specimens obtained at different times during the acute phase of illness of the same patient were included in the temporal analysis (Figure). MAC-ELISA results for acute-phase serum specimens of the 33 remaining 1999 case-patients who were not followed longitudinally were also included (Figure). When case-patients became serologically negative for WNV-reactive IgM, they were not subsequently resampled. Since the timing of the sequential bleeds depended on the availability of the patients, not all patients were sampled at all follow-up time intervals. Results from the ELISA testing were expressed as a positive-to-negative (P/N) ratio of observed A_450nm_ (MAC-ELISA) or A_405nm_ (IgG ELISA) as described (*16*,*20*). In these tests, P/N ratios >3.0 were considered positive, and P/N ratios >2.0 and <3.0 were considered equivocal, requiring additional laboratory testing. The MAC-ELISA and IgG ELISA were run in triplicate for each specimen.

**Figure:**
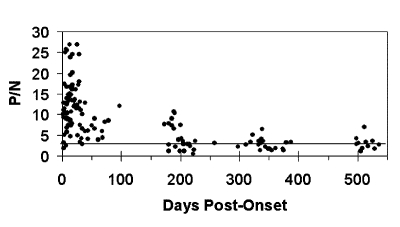
Scatterplot of anti–West Nile virus immunoglobulin M positive-to-negative (P/N) values of individual serum specimens over time. Dotted line represents P/N=3.0 cut-off.

Scatter plot analysis of the IgM activity of all tested specimens showed a typical WNV-reactive IgM response (Figure), similar to that seen with human infections with that virus (*19*). The IgM activity peaked within the first 20 days after onset. Of the 29 case-patients followed for long-term antibody activity, 22 had serum specimens obtained approximately 200 days after onset, 21 had serum specimens obtained 200–400 days after onset, and 12 had serum specimens obtained >500 days after onset (Table 1). Of the 22 specimens obtained approximately 200 days after onset, 14 (64%) were WNV-reactive IgM-positive (P/N values >3.0; range 3.0–10.8; average 6.3). An additional four specimens had equivocal results (P/N values from 2.0 to 2.99), which would require additional laboratory testing. In total, 18 (82%) of 22 specimens had either positive or equivocal results at approximately 200 days after onset.

**Table 1:** IgM and IgG P/N values of WNV virus patient serum samples by days after onset^a^

Case no.	Sample	Days after onset	WNV IgM	WNV IgG
1	S1	12	14.3	2.0
	S2	197	3.9	7.8
	S3	340	3.5	8.2
	S4	498	2.8	nd^a^
2	S1	97	12.0	5.3
	S2	378	3.2	nd
3	S1	72	8.2	5.0
	S2	186	7.3	7.0
	S3	388	1.0	nd
4	S1	27	17.3	3.4
	S2	311	2.7	6.4
5	S1	31	11.0	5.5
	S2	215	2.8	7.9
	S3	374	1.3	nd
6	S1	39	5.9	2.8
	S2	173	7.6	3.9
	S3	332	3.5	4.3
	S4	502	3.1	nd
7	S1	29	11.5	4.8
	S2	189	6.7	6.3
	S3	332	2.7	6.2
	S4	536	2.8	nd
8	S1	3	3.3	6
	S2	15	10.7	5.0
	S3	30	nd	5.3
	S4	43	4.1	5.4
	S5	204	3.5	6.2
	S6	343	2.2	7.0
	S7	508	1.8	nd
9	S1	21	13.6	4.4
	S2	199	1.7	3.2
10	S1	55	9.0	3.7
	S2	189	10.8	6.0
11	S1	17	24.6	5.2
	S2	255	3.6	4.3
	S3	336	3.1	7.5
	S4	512	6.9	nd
12	S1	11	13.4	5.3
	S2	13	15.0	5.6
	S3	24	11.4	5.8
	S4	533	1.8	nd
13	S1	8	16.7	3.4
	S2	202	4.2	nd
14	S1	16	7.8	2.4
	S2	30	3.5	5.6
	S3	349	1.7	7.0
15	S1	17	17.2	5.6
	S2	192	2.2	6.3
	S3	335	1.4	6.8
16	S1	7	14.0	2.0
	S2	29	24.5	3.7
	S3	338	6.5	6.6
17	S1	79	8.5	5.7
	S2	200	7.5	4.5
	S3	337	4.1	4.6
	S4	525	3.6	nd
18	S1	50	7.3	4.8
	S2	55	6.6	3.3
	S3	68	4.4	nd
	S4	207	2.9	7.3
	S5	505	1.3	nd
19	S1	4	10.5	4.0
	S2	14	7.3	4.1
	S3	355	1.3	nd
20	S1	29	18.0	3.1
	S2	257	3	8.8
	S3	373	1.7	nd
21	S1	1	10.1	3.6
	S2	7	9.2	3.5
	S3	180	1.2	2.8
22	S1	6	25.7	2.4
	S2	185	9.1	6.9
	S3	319	3.4	8.2
	S4	497	4.2	Nd
23	S1	25	12.3	5.5
	S2	204	3.0	7.2
	S3	529	1.7	nd
24	S1	4	11.4	3.2
	S2	8	9.1	3.5
	S3	14	19.6	2.7
	S4	212	2.9	6.3
	S5	361	1.9	nd
25	S1	9	9.1	4.8
	S2	34	6.7	5.2
	S3	223	1.5	5.0
26	S1	10	14.8	2.3
	S2	22	16.1	3.7
	S3	191	10.4	3.2
	S4	387	3.4	nd
	S5	514	5.2	nd
27	S1	3	11.1	3.7
	S2	180	7.8	5.8
	S3	322	5.1	8.3
	S4	518	2.4	nd
28	S1	26	4.9	6.0
	S2	32	4.3	5.6
	S3	298	2.2	nd
29	S1	217	2.4	6.7
	S2	351	1.8	8.1

^a^ Ig, immunoglobulin; P/N, positive-to-negative; WNV, West Nile virus nd, not done.

Of the 21 serum specimens obtained approximately 300–400 days after onset, 9 (43%) were WNV-reactive IgM-positive (P/N values >3.0; range 3.1–6.5; average 4.0). An additional four specimens had equivocal results (P/N values of 2.2, 2.2, 2.7, and 2.7), which would require additional laboratory testing. In total, 13 (62%) of 21 specimens had either positive or equivocal results at 300–400 days after onset.

Of the 12 serum specimens obtained approximately >500 days after onset, 5 (42%) were WNV-reactive IgM-positive (P/N values ≥3.0; range 3.1–6.9; average 4.6). An additional two specimens had equivocal results (P/N value 2.8), which would require additional laboratory testing. In total, 7 (58%) of 12 specimens had either positive or equivocal results at approximately 500 days after onset, with the latest positive specimen having been drawn 525 days (17.5 months) after onset (P/N = 3.6).

As expected, most of the 29 case-patients had positive WNV-reactive IgG results (P/N ≥3.0; range 3.2–8.8; average 6.3) in their last tested serum specimen. The one patient whose P/N was <3.0 had a P/N of 2.8, which means that all specimens would probably be considered WNV antibody-positive or at least require additional laboratory testing. Although we did not follow longitudinally the WNV-reactive IgM activity in the CSF of these case-patients, the latest WNV-positive CSF specimen ever submitted to Centers for Disease Control and Prevention for diagnostic testing from a laboratory-confirmed WNV human infection was obtained at 47 days after onset (data not shown).

The percent of patients with detectable IgM antibodies at 9 months differed by age (Table 2), with 56% of those ≥65 years of agebeing positive compared to 44% of those <65 years; the difference was not statistically significant. No difference existed in the percentage positive at 1 year by sex or initial clinical syndrome, with 18% of encephalitis patients being positive, compared with 0% of meningitis patients. Patients with acute IgM P/N ratios above the median appeared to be more likely to remain positive over time than those with acute P/N ratios below the median.

**Table 2:** Percent of persons with IgM-positive serology by months after onset^a^

	Number WNV IgM positive/total (%)				
	Acute (n=27)	3 months (n=15)	6 months (n=23)	9 months (n=23)	12 months (n=18)
**Age**					
<65 yrs	9/9 (100)	6/6 (100)	6/9 (67)	4/8 (50)	0/5 (0)
+65 yrs	18/18 (100)	9/9 (100)	9/14 (64)	5/15 (33)	2/13 (15)
**Sex**					
Male	13/13 (100)	8/8 (100)	8/11 (73)	3/10 (30)	1/9 (11)
Female	14/14 (100)	7/7 (100)	7/12 (58)	6/13 (46)	1/9 (11)
**Clinical syndrome**					
Encephalitis	16/16 (100)	8/8 (100)	8/13 (62)	5/13 (39)	2/11 (18)
Meningitis	11/11 (100)	7/7 (100)	7/10 (70)	4/10 (40)	0/7 (0)
**Baseline IgM^b^**					
<Median P/N	14/14 (100)	6/6 (100)	6/11 (55)	3/12 (25)	0/10 (0)
>median P/N	13/13 (100)	9/9 (100)	9/12 (75)	6/11 (55)	2/8 (25)

^a^WNV, West Nile virus; Ig, immunoglobulin; P/N, positive-to-negative; P/N ratio > 3.0. ^b^Median P/N = 11.4.

## Conclusions

Identification of a recent human infection with WNV is a important event that usually triggers public health alerts, mosquito control measures, and media attention. Because laboratory tests for the presence of WNV-reactive IgM are used to identify such infections, they should be conducted properly, and the test results must be interpreted accurately. Proper interpretation criteria include considering clinical context (encephalitis or meningitis), previous travel history, flaviviral vaccination history, and evidence of previous and current WNV activity in the region. Cumulatively during 1999–2000, onset dates for human WNV disease cases in the United States ranged from early July to early December, roughly indicating a 5-month transmission season and a 7-month nontransmission season (*7*,*8*). Based on the serologic results presented here, approximately 50% of persons with an acute WNV infection of the central nervous system would be expected to have persistent IgM antibody for >8 months. The presence of WNV-reactive IgM in serum alone, therefore, is not necessarily diagnostic of an acute WNV infection. These IgM results with human WNV infections concur with the previously published results of human JEV infections and suggest that the temporal characteristics of the human antibody response to related neurotropic flaviviruses (e.g., WNV, JEV, St. Louis encephalitis virus, and Murray Valley encephalitis virus) are similar (*17*).

Given the low incidence of indigenously acquired neurotropic flavivirus infections in the United States, however, this similarity would seem to be more of a theoretical concern than a practical one (i.e., the chance of a person in the United States acquiring WNV encephalitis or meningitis during a given transmission season, maintaining a significant level of virus-specific IgM activity over the ensuing 8–12 months, and then again developing a viral encephalitis, meningitis, or being re-exposed to WNV during the subsequent transmission season is highly unlikely). Therefore, when evaluating a patient with acute viral encephalitis acquired in the United States, a positive serum test for IgM antibody to WNV would be expected to have a high predictive value, particularly during July to December, and especially when additional evidence exists of current epizootic or epidemic WNV activity in the area.

Nevertheless, especially in areas where WNV is known to have circulated previously or has an extended transmission season (e.g*.*, Florida), suspected cases of acute WNV disease of the central nervous system should be confirmed by the demonstration of WNV-reactive IgM in CSF, the development of WNV-specific IgG antibody in convalescent-phase serum (ideally, by demonstrating a fourfold change in neutralizing antibody titer between the acute and convalescent phases), or both. We have determined empirically that the cross-reactivity of the WNV-reactive IgM appears to be less than that of Saint Louis encephalitis virus-reactive human IgM; therefore, conducting concurrent tests with the other indigenous neurotropic flaviviruses, which now coexist with WNV in some parts of the United States (*21*), is also important.

The observed apparent-to-inapparent WNV infection ratio in the United States is approximately 1:140, which indicates that a large group of persons with subclinical WNV infections exist (*22*). While the serologic results presented here are for patients with neuroinvasive WNV disease and may not be generalizable to patients with clinically mild or inapparent WNV infection, the possibility exists that many mild (and probably undiagnosed) infections occur and further emphasizes the need for careful laboratory assessment before a diagnosis of acute WNV infection.
